# PEPIS: A Pipeline for Estimating Epistatic Effects in Quantitative Trait Locus Mapping and Genome-Wide Association Studies

**DOI:** 10.1371/journal.pcbi.1004925

**Published:** 2016-05-25

**Authors:** Wenchao Zhang, Xinbin Dai, Qishan Wang, Shizhong Xu, Patrick X. Zhao

**Affiliations:** 1 Plant Biology Division, Samuel Roberts Noble Foundation, Ardmore, Oklahoma, United States of America; 2 School of Agriculture and Biology, Shanghai Jiao Tong University, Shanghai, People’s Republic of China; 3 Department of Botany and Plant Sciences, University of California, Riverside, Riverside, California, United States of America; University of Canterbury, NEW ZEALAND

## Abstract

The term epistasis refers to interactions between multiple genetic loci. Genetic epistasis is important in regulating biological function and is considered to explain part of the ‘missing heritability,’ which involves marginal genetic effects that cannot be accounted for in genome-wide association studies. Thus, the study of epistasis is of great interest to geneticists. However, estimating epistatic effects for quantitative traits is challenging due to the large number of interaction effects that must be estimated, thus significantly increasing computing demands. Here, we present a new web server-based tool, the Pipeline for estimating EPIStatic genetic effects (PEPIS), for analyzing polygenic epistatic effects. The PEPIS software package is based on a new linear mixed model that has been used to predict the performance of hybrid rice. The PEPIS includes two main sub-pipelines: the first for kinship matrix calculation, and the second for polygenic component analyses and genome scanning for main and epistatic effects. To accommodate the demand for high-performance computation, the PEPIS utilizes C/C++ for mathematical matrix computing. In addition, the modules for kinship matrix calculations and main and epistatic-effect genome scanning employ parallel computing technology that effectively utilizes multiple computer nodes across our networked cluster, thus significantly improving the computational speed. For example, when analyzing the same immortalized F2 rice population genotypic data examined in a previous study, the PEPIS returned identical results at each analysis step with the original prototype R code, but the computational time was reduced from more than one month to about five minutes. These advances will help overcome the bottleneck frequently encountered in genome wide epistatic genetic effect analysis and enable accommodation of the high computational demand. The PEPIS is publically available at http://bioinfo.noble.org/PolyGenic_QTL/.

## Author Summary

A new mixed-model was recently developed for mapping DNA regions that are associated with variations in observable traits, known as quantitative trait loci. This model incorporates multiple polygenic covariance genetic structures and has been used to successfully predict yield in hybrid rice. However, quantitatively examining interactions between multiple genetic elements, i.e. epistatic effects, across the entire genome is computationally demanding, which greatly hinders real-world applications of the model, especially in evaluating large populations and multiple genetic markers. In order to overcome these computational challenges, we developed a tool known as the PEPIS—Pipeline for estimating EPIStatic genetic effects in genome wide. We implemented the PEPIS in efficient C/C++ programming language and successfully incorporated several highly efficient open-source mathematical operation and optimization libraries to perform the computational demanding mathematical matrix operations required for these types of analyses. Furthermore, the computational demand is distributed across multiple nodes on our networked Linux computing cluster in order to increase the speed of analyses. Our PEPIS tool will thus help overcome the bottleneck in genetic epistasis analysis.

This is a *PLOS Computational Biology* Software Article.

## Introduction

Epistasis, the interaction among multiple genetic loci, contributes significantly to phenotypic variation associated with the expression of polygenic complex traits. Epistatic effects have emerged as a possible explanation for ‘missing heritability,’ that is, marginal genetic effects that cannot be accounted for in a genome-wide association study (GWAS). Examinations of epistatic effects may also enhance understanding of the hierarchical architecture of gene interactions and genetic pathways [1].

Depending on the characteristics of a given study population, variations in the epistatic effects associated with a quantitative trait can be classified based on a number of different variance components, such as additive by additive, additive by dominance, dominance by additive, and dominance by dominance, for example [2]. The relative importance of each variance component usually varies across different traits.

GWAS has played important roles in the identification of possible connections between single-nucleotide polymorphisms (SNPs) and various diseases [3]. However, a number of studies that have identified genetic susceptibility factors failed to account for the effects of interactions between multiple genetic loci [4,5], which may help explain why the genetic variability of individual genes by GWAS can only explain ~40% variation of psychiatric disorders [4]. This inability to provide complete genetic explanations gives support to the concept of missing heritability. Careful analyses of epistatic effects may help close the gap in our understanding of missing heritability [4,6].

Obtaining a thorough understanding of the genetic architecture of a quantitative trait is particularly important in plant and animal breeding in order to develop optimal breeding strategies and obtain maximum genetic gains [7]. To date, however, most applications of quantitative genetics in plant and animal breeding have involved additive models based on the assumption that non-additive genetic effects are not important [2,8]. Unfortunately, breeding populations rarely exhibit such ideal conditions, leading to confounding of genetic values associated with additive and non-additive effects. Under the non-ideal conditions of real-world practice, a large proportion of the variance resulting from interactions between alleles (i.e., dominance and epistasis) may appear as additive variance [9].

A considerable amount of attention has been given to mapping of quantitative trait loci (QTL) to examine epistatic effects [10–15]. Xu et al. recently proposed a new mixed-model method for QTL mapping that incorporates multiple polygenic covariance structures [16]. In this model, genome-wide markers are used to initially estimate six different kinship matrices. Then, the total genetic variance was partitioned into six variance components. Each kinship matrix corresponds to one of the variance components: additive, dominance, additive by additive, dominance by dominance, additive by dominance, and dominance by additive. The six different kinship matrices, along with the six estimated polygenic variances, are then examined by polygenic QTL mapping. This model has been used to successfully predict the performance of hybrid rice [17] using released rice SNP data [18,19]. However, the large number of interaction effects to be estimated poses a significant obstacle in epistatic-effect QTL mapping. Although the ~270,000 original SNPs in the study by Xu et al. [16] were eventually converted into 1,619 synthetic markers (bins) [18], complete analysis of all 278 immortalized F2 (IMF2) individuals using their prototype R (www.r-project.org) scripts would take more than one month.

Motivated by the challenge presented by the tremendous computational demand associated with epistatic QTL mapping, we conducted a thorough investigation of the model developed by Xu et al. and re-implemented the model algorithm using C/C++, resulting in the development of a web server-based tool named PEPIS (Pipeline for estimating EPIStatic genetic effects). The PEPIS employs parallelized kinship matrix calculations and main- and epistatic-effect genome scanning. Large computational analyses are divided and allocated to computational nodes on our networked Linux clusters. Furthermore, an open-source C++ linear algebra library, Armadillo [20], was utilized for mathematical matrix operations. The benefit of these strategies is a substantial reduction in computational time. Using the released IMF2 population rice SNP data, PEPIS reported the same result at each step when compared with the original prototype script developed by Xu., but reduced the whole analysis time from more than one month to about five minutes. Herein, we believe that this is a remarkable achievement that has overcome the bottleneck in epistatic analysis and thus empowers the high computational demanding of epistatic QTL mapping.

### Genetic Model and Statistical Analysis Method

As the genetic model and statistical method proposed by Xu et al. [16,17] served as the basis for the development of the PEPIS, a brief review of their work is in order. First, the genotype of individual *j* in bin *k* is numerically coded into two variables, as follows:

$$Z_{jk}=\{\begin{array}{ccc} +1 & \begin{array}{cc} for & A \end{array} & \\ 0 & \begin{array}{cc} for & H \end{array} & and\\ -1 & \begin{array}{cc} for & B \end{array} & \end{array}\;W_{jk}=\{\begin{array}{ccc} 0 & \begin{array}{cc} for & A \end{array} & \\ 1 & \begin{array}{cc} for & H \end{array} & \\ 0 & \begin{array}{cc} for & B \end{array} & \end{array}$$ (1)

where *Z*_*jk*_ and *W*_*jk*_ represent additive and dominance indicators, respectively, and *A* (the first homozygote), *H* (heterozygote), and *B* (the second homozygote) indicate the three genotypes.

Let *y* be an *n*×1 vector for the quantitative trait values of all *n* individuals. It can be expressed by the following complete epistatic model for *m* bins,

$$\begin{array}{l} y=X\beta +\sum_{k=1}^{m}Z_{k}a_{k}+\sum_{k=1}^{m}W_{k}d_{k}+\sum_{k=1}^{m-1}\sum_{k^{'}=k+1}^{m}(Z_{k}\#Z_{k^{'}}){\left(aa\right)}_{kk^{'}}+\sum_{k=1}^{m-1}\sum_{k^{'}=k+1}^{m}(Z_{k}\#W_{k^{'}}){\left(ad\right)}_{kk^{'}}+\\ \sum_{k=1}^{m-1}\sum_{k^{'}=k+1}^{m}(W_{k}\#Z_{k^{'}}){\left(da\right)}_{kk^{'}}+\sum_{k=1}^{m-1}\sum_{k^{'}=k+1}^{m}(W_{k}\#W_{k^{'}}){\left(dd\right)}_{kk^{'}}+\varepsilon \end{array}$$ (2)

where *Xβ* represents non-genetic effects and *a*_*k*_ and *d*_*k*_ represent additive and dominance effects, respectively, for bin *k*. The terms (*aa*)_*kk*'_, (*ad*)_*kk*'_, (*da*)_*kk*'_, and (*dd*)_*kk*'_ represent additive by additive, additive by dominance, dominance by additive, and dominance by dominance effects, respectively, for bins *k* and *k*'.

In this model, the terms $\sum_{k=1}^{m}Z_{k}a_{k}$ and $\sum_{k=1}^{m}W_{k}d_{k}$ describe polygenic main effects; whereas $\sum_{k=1}^{m-1}\sum_{k^{'}=k+1}^{m}(Z_{k}\#Z_{k^{'}}){\left(aa\right)}_{kk^{'}}$, $\sum_{k=1}^{m-1}\sum_{k^{'}=k+1}^{m}(Z_{k}\#Z_{k^{'}}){\left(ad\right)}_{kk^{'}}$, $\sum_{k=1}^{m-1}\sum_{k^{'}=k+1}^{m}(Z_{k}\#Z_{k^{'}}){\left(da\right)}_{kk^{'}}$, and $\sum_{k=1}^{m-1}\sum_{k^{'}=k+1}^{m}(Z_{k}\#Z_{k^{'}}){\left(dd\right)}_{kk^{'}}$ describe polygenic epistatic effects.

When the genetic effects are treated as normally distributed random variables with a mean of zero and a common variance across all markers or marker pairs, we have a mixed-model results. Let $\sigma_{a}^{2}$, $\sigma_{d}^{2}$, $\sigma_{aa}^{2}$, $\sigma_{ad}^{2}$, $\sigma_{da}^{2}$, and $\sigma_{dd}^{2}$ be the variance components associated with each of the six types of genetic effects. The expectation of *y* is *E*(*y*) = *Xβ*, and the variance matrix of *y* is

$$Var(y)=K_{a}\sigma_{a}^{2}+K_{d}\sigma_{d}^{2}+K_{aa}\sigma_{aa}^{2}+K_{ad}\sigma_{ad}^{2}+K_{da}\sigma_{da}^{2}+K_{dd}\sigma_{dd}^{2}+I\sigma^{2}$$ (3)

where *σ*^2^ represents the residual error variance.

Each *K* matrix corresponds to a marker-generated kinship matrix, and its value can be calculated utilizing the formulas reported by Xu et al. [16]. Genetic similarities between all of the individuals in the sample can be assessed using the corresponding matrices. The variance components can be estimated using standard mixed-model procedures in conjunction with the marker-generated kinship matrices using the restricted maximum likelihood method (REML).

Two likelihood values are needed, one associated with the alternative hypothesis, *H*_*1*_, and the other with the null hypothesis, *H*_*0*_. The likelihood ratio test (LRT) can be used as an indicator of the degree of deviation of *H*_*1*_ from *H*_*0*_. In the original prototype script code developed by Xu et al., the restricted maximum-likelihood estimation (REML) method was employed to estimate the variance components and the vector $\theta =[\sigma_{a}^{2},\sigma_{d}^{2},\sigma_{aa}^{2},\sigma_{ad}^{2},\sigma_{da}^{2},\sigma_{dd}^{2}]$. The REML log-likelihood function is defined below

$$L(\theta )=\frac{1}{2}\ln\left|V\right|-\frac{1}{2}y^{T}P_{X}y-\frac{1}{2}\ln\left|X^{T}V^{-1}X\right|$$ (4)

where *P*_*X*_ = *V*^−1^−*V*^−1^*X*(*X*^*T*^*V*^−1^*X*)^−1^*X*^*T*^*V*^−1^.

The variance matrix shown in Eq (3) can thus be rewritten as

$$Var(y)=(K_{a}\lambda_{a}+K_{d}\lambda_{d}+K_{aa}\lambda_{aa}+K_{ad}\lambda_{ad}+K_{da}\lambda_{da}+K_{dd}\lambda_{dd}+I)\sigma^{2}$$ (5)

where $\lambda_{a}=\sigma_{a}^{2}/\sigma^{2}$, $\lambda_{d}=\sigma_{d}^{2}/\sigma^{2}$, $\lambda_{ad}=\sigma_{aa}^{2}/\sigma^{2}$, $\lambda_{ad}=\sigma_{ad}^{2}/\sigma^{2}$, $\lambda_{da}=\sigma_{da}^{2}/\sigma^{2}$, and $\lambda_{dd}=\sigma_{dd}^{2}/\sigma^{2}$ are variance ratios. The six polygenic variance ratios are then collected in a vector **λ** = [*λ*_*a*_,*λ*_*d*_,*λ*_*aa*_,*λ*_*aa*_,*λ*_*da*_,*λ*_*dd*_]. Let **K** = [*K*_*a*_,*K*_*d*_,*K*_*aa*_,*K*_*ad*_,*K*_*da*_,*K*_*dd*_]^*T*^, then we have *Var*(*y*) = *V* = (**Kλ** + *I*)*σ*^2^.

Given the six variance ratios, a complete polygenic structure for a target quantitative trait can be examined in detail. One-dimensional (1D) genome scanning for main effects and two-dimensional (2D) genome scanning for epistatic effects can be employed to estimate individual marker/bin (main) effects and marker/bin pair interaction (epistatic) effects. Here, the individual main and epistatic effects correspond to two and four degrees of freedom LRT test, respectively. In theory, the four degrees of freedom LRT test needs four distinguishable genotypic forms. However, it is rare but possible that only three or less distinguishable genotypic forms occur for a marker/bin pair. This will lead to less than four degrees of freedom for the LRT test for epistatic effects. If we adopt a random model approach, there will be no problem for parameter estimation even if less than four genotypic forms exist. In other words, the random model does not depend on full rank of the design matrix.

## Design and Implementation

### Design Overview

We developed the PEPIS for rapid epistatic QTL mapping analyses. The PEPIS is composed of two primary sub-pipelines. Sub-pipeline 1 is used for kinship matrix calculations, and sub-pipeline 2 is used for polygenic QTL mapping and integrates three related analysis modules: one for polygenic variance component analysis, another for genome scanning for main effects, and the third module for genome scanning for epistatic effects. The four modules (sub-pipeline 1 and the three modules of sub-pipeline 2) are designated ‘km_cal’, ‘pc_anal’, ‘gs_main’, and ‘gs_epis’, respectively. All the modules were coded in C/C++ and compiled into four separate executable command line programs. Several perl and cshell script files were then developed to function as a wrapper to streamline the complete pipeline.

**Fig 1:**
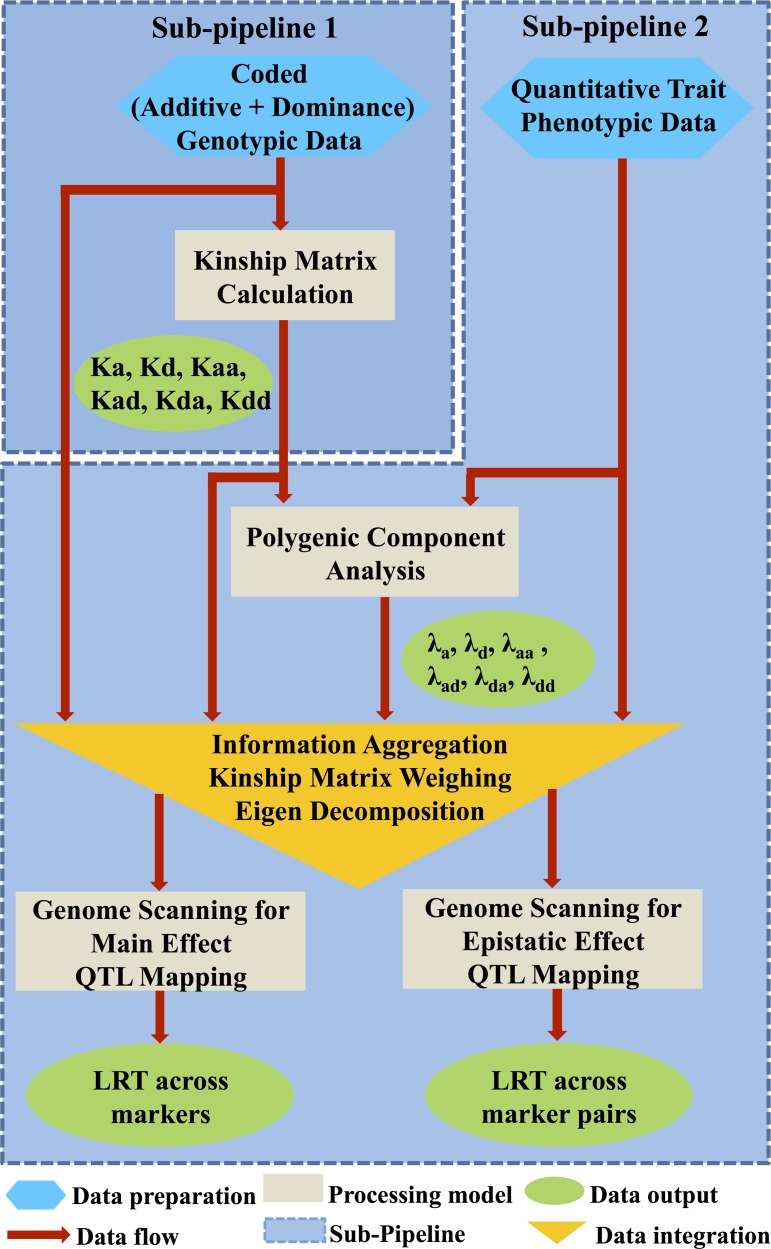
Flowchart illustrating polygenic QTL mapping using the PEPIS. The PEPIS is divided into two parts: sub-pipeline 1, used for the six polygenic kinship matrix calculations, and sub-pipeline 2, used for the six polygenic component ratio estimations and further genome scanning for main and epistatic genetic effects.

When coded genotype data are provided, module km_cal calculates and delivers the corresponding kinship matrices. When phenotypic quantitative trait data are provided, module pc_anal estimates and delivers the six polygenic variances utilizing both the quantitative trait data and the available kinship matrices. Following the performance of various information aggregation procedures, including kinship matrix weighing and matrix eigen decomposition, modules gs_main and gs_epis calculate and return 1D LRT values for all markers (bins) and 2D LRT values for all marker (bin) pairs, respectively. **Fig 1** illustrates the overall flow of polygenic QTL mapping analyses in the PEPIS.

To increase the flexibility of analyses, the PEPIS allows users to run only a portion of the pipeline according to the input data and user-configured parameters (e.g., users can perform only kinship matrix calculations and polygenic variance component analyses, or they can perform only kinship matrix calculations or even only calculate some of the kinship matrices). Such configuration flexibility allows users to utilize specific PEPIS-generated information with their own statistical genetics models.

### Computational Implementation of Mathematical Matrix Operations and LRT Optimization

A complete analysis requires processing of a large volume of genotypic matrix data and necessarily involves complex mathematical matrix operations, such as transposition, inversion, determinant calculation, and eigen decomposition, which can be easily prototyped in R or MATLAB (www.mathworks.com) without considering speed. However, as our goal in developing the PEPIS was to speed up the analysis of genotypic data to identify epistatic effects, we utilized C/C++ and a C++ linear algebra package, thus enabling highly efficient processing.

Because C++ is the programming language used in this project, we chose the C++-based linear algebra library Armadillo [20], which is an open-source program that provides a good balance between speed and ease of use. Furthermore, Armadillo’s API syntax was deliberately designed to be similar to MATLAB. A comparison of performance indicated that Armadillo is substantially faster than both MATLAB and previously developed C++ libraries such as IT++ and Newmat (http://www.robertnz.net/nm_intro.htm) [20]. Based on these advantages, we incorporated Armadillo source codes (http://arma.sourceforge.net/) for performing the mathematical matrix operations in the PEPIS.

Armadillo utilizes the BLAS (Basic Linear Algebra Subprograms, http://www.netlib.org/blas/) and LAPACK (Linear Algebra Package, http://www.netlib.org/lapack) for low-level computations such as basic vector and matrix and linear algebra operations. As such, it was necessary to incorporate both of these packages in the PEPIS.

LRT analysis requires two maximum-likelihood estimations, one of the alternative hypothesis *H*_*1*_, and one of the null hypothesis, *H*_*0*_. Furthermore, the maximum-likelihood estimation is even more computationally demanding, as it is essentially a bound constrained optimization procedure. The R environment provides the ‘optim()’ function and the ‘L-BFGS-B’ algorithm [21,22] options for users to perform specific optimizations. To develop the PEPIS in C/C++, we therefore adopted and incorporated the L-BFGS-B open-source codes in C (http://users.iems.northwestern.edu/~nocedal/lbfgsb.html) for the optimization sub-routine, which is also utilized as an optimization tool in the R environment.

### Parallel Strategy for Distributed High-Performance Computing

As our goal was to facilitate rapid epistatic QTL mapping, we first needed to analyze the computational complexity of the model and resolve the fundamental problems associated with the computationally demanding nature of these analyses. If we suppose that the number of individuals is represented by *n* and the number of markers/bins by *m*, then the number of total genetic effects is 2*m*+4*C*(*m*,2) = 2*m*^2^. In the formulas for calculating the six kinship matrices [16], each kinship matrix is a square matrix of size *n*×*n* and matrix cell value *K*[*i*,*j*] = *K*[*j*,*i*]. Considering this symmetric feature, the multiplication time for *K*_*a*_ and *K*_*d*_ is $\frac{mn(n+1)}{2}$, which is on the order of *O*(*mn*^2^). The multiplication time for *K*_*aa*_, *K*_*ad*_, *K*_*da*_, and *K*_*dd*_ is $\frac{m(m-1)n(n+1)}{4}$, which is on the order of *O*(*m*^2^*n*^2^). These estimations clearly demonstrate the enormity of the multiplication demand associated with kinship matrix calculations, especially when both the individual and marker/bin numbers are large. However, the procedure used to calculate each matrix cell value is the same; thus, all $\frac{n(n+1)}{2}$ loops for matrix cell calculation can be parallelized.

The polygenic variance component analysis module needs essentially only one optimization for a seven-parameter log-likelihood estimation. The main-effects genome scanning module requires *m* times two degrees of freedom LRT estimation, and the epistatic-effects genome scanning module requires $\frac{m(m-1)}{2}$ times four degrees of freedom LRT estimation. Similarly, the procedure to estimate the LRT is the same, so the *m* times two degrees of freedom LRT estimation and $\frac{m(m-1)}{2}$ times four degrees of freedom LRT estimation can also be parallelized.

As demonstrated above, the computationally intensive modules for kinship matrix calculations and genome scanning for main and epistatic effects can be parallelized to increase the speed and efficiency of the analyses. The strategy utilized in the PEPIS for parallel high-performance distributed computing is summarized in **Table 1**. Currently, the PEPIS is configured to efficiently utilize ~500 central processing unit (CPU) nodes in our Linux clusters for parallel computations.

**Table 1:** Summary of parallel strategy in the PEPIS for increasing analysis speed.

Processing Model	Computation complexity description	Repetitive parallelizable	Allocated job for each CPU node with p parallelizable CPUs
		calculation unit	
Kinship matrix calculation	**n(n+1)**/**2** loops for 6 kinship matrix cell calculations. **m(m−1)n(n+1)**+**mn(n+1)** times multiplication	6 kinship matrix cell calculations	**n(n+1)**/**2p** loops for 6 matrix cell calculations
Genome scanning for main effects	**m** times 2 degrees of freedom LRT estimation	2 degrees of freedom LRT estimation	**m/p** times 2 degrees of freedom LRT estimation
Genome scanning for epistatic effects	**m(m−1)/2** times 4 degrees of freedom LRT estimation	4 degrees of freedom LRT estimation	**m(m−1)/2p** times 4 degrees of freedom LRT estimation

## Results

The PEPIS is a web-based program developed in C/C++ to facilitate efficient and rapid epistatic QTL mapping. We verified that the PEPIS could meet our performance expectations by analyzing the same IMF2 rice population genotypic and field phenotypic trait data sets examined by Xu et al. [16].

### Field Data and IMF2 Population

The IMF2 population described by Hua et al. [23,24] consisted of 360 crosses made by random matches of 240 recombinant inbred lines (RILs) derived by single-seed descent from a cross between the Zhenshan 97 and Minghui 63 rice hybrids. Field data pertaining to yield (YIELD), number of tillers per plant (TILLER), number of grains per panicle (GRAIN), and thousand-grain weight (KGW) were collected during the 1998 and 1999 rice growing seasons from replicated field trials on the Huazhong Agricultural University Experimental Farm in Wuhan, China. Over 270,000 high-density SNP markers were used to infer recombination breakpoints (crossovers), which were then used to construct a total of 1,619 bins [18]. The bins were treated as “new markers” for association studies. The bin map was constructed by genotyping the RIL population sequences [18,19]. Of the 360 crosses, only 278 were available in both phenotypes and bin genotypes. Therefore, the bin genotype data were stored in an *n*×*m* = 278×1,619 matrix. The Zhenshan 97 genotype was coded as ‘A,’ the Minghui 63 genotype as ‘B,’ and the heterozygote as ‘H.’ We downloaded the genotype and phenotype data from the website specified by Zhou et al. [25].

### Submission of Representative Case Analysis Data to the PEPIS and Results Returned

The coded additive and dominance genotypic data were stored as two *n*×*m* = 278×1,619 matrices and then submitted to the PEPIS. Simultaneously, the YIELD, KGW, GRAIN, and TILLER quantitative phenotypic data were stored as a *n*×1 = 278×1 column vector for each trait and submitted to the PEPIS. Upon clicking the submit icon, the PEPIS allocates the entire processing job to the available computer nodes distributed across our networked Linux clusters. Once all of the distributed computing jobs are completed, the results are returned as a pop-up page, and users are given the option to download the results from each analysis step. Using the same IMF2 population rice data, the PEPIS returns the same result at each step when compared with the original prototype script developed by Xu et al. [16], but reduced the whole analysis time from more than one month to about five minutes. The significant difference in analysis time demonstrates that the PEPIS is capable of performing large-scale epistatic QTL mapping based on data from large numbers of individuals and markers/bins.

**Fig 2:**
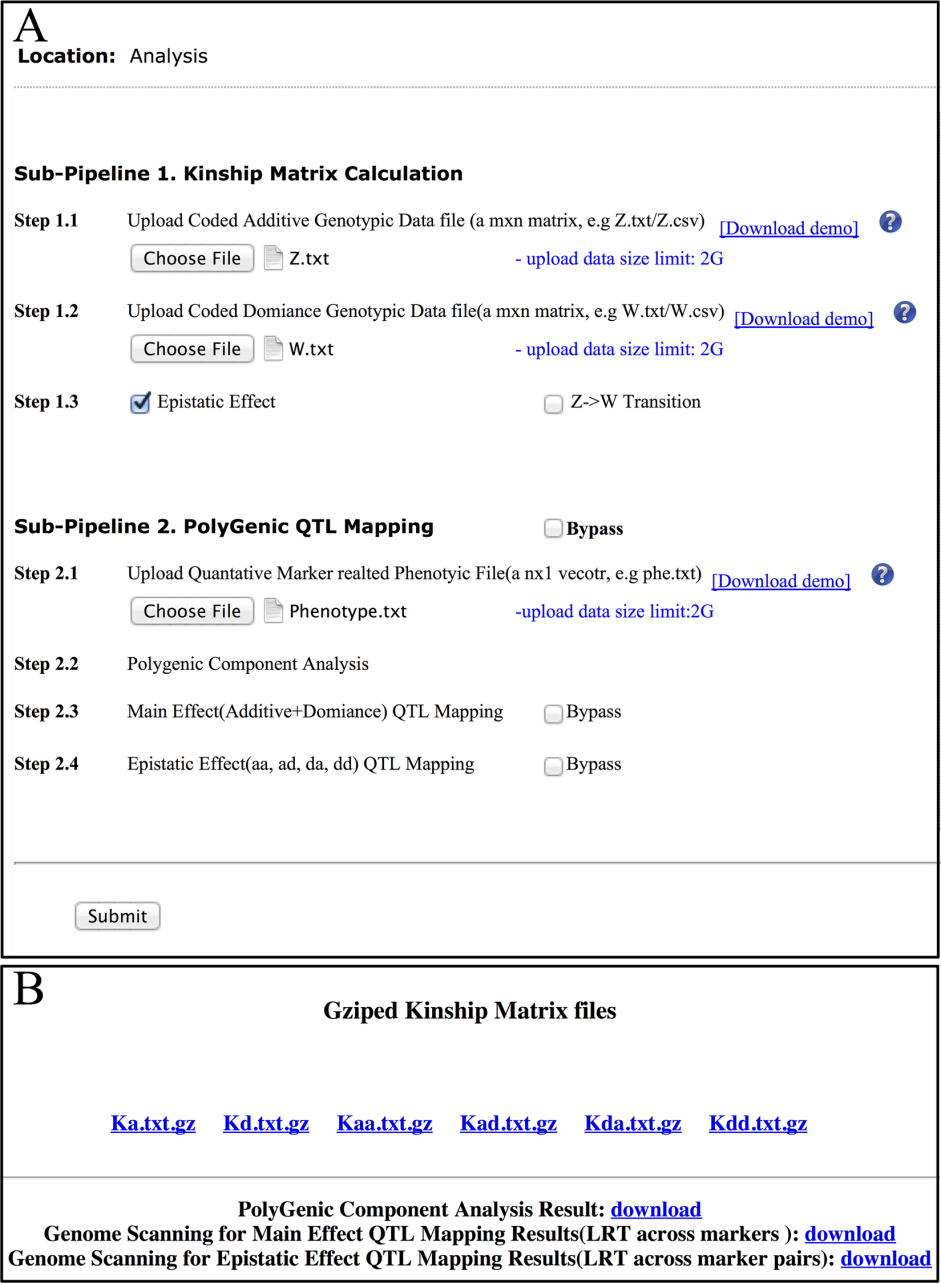
PEPIS user interfaces. (A) Data submission and (B) Results return.

The user interface for data submission and return of results is shown in **Fig 2**. The results returned include the six ‘gzip’ files corresponding to the six polygenic kinship matrices, as well as three '.txt' files corresponding to the results of polygenic variance component analysis and genome scanning for main and epistatic effects. The analysis results are explained in detail below.

### PEPIS Results and Biological Implications

**Fig 3:**
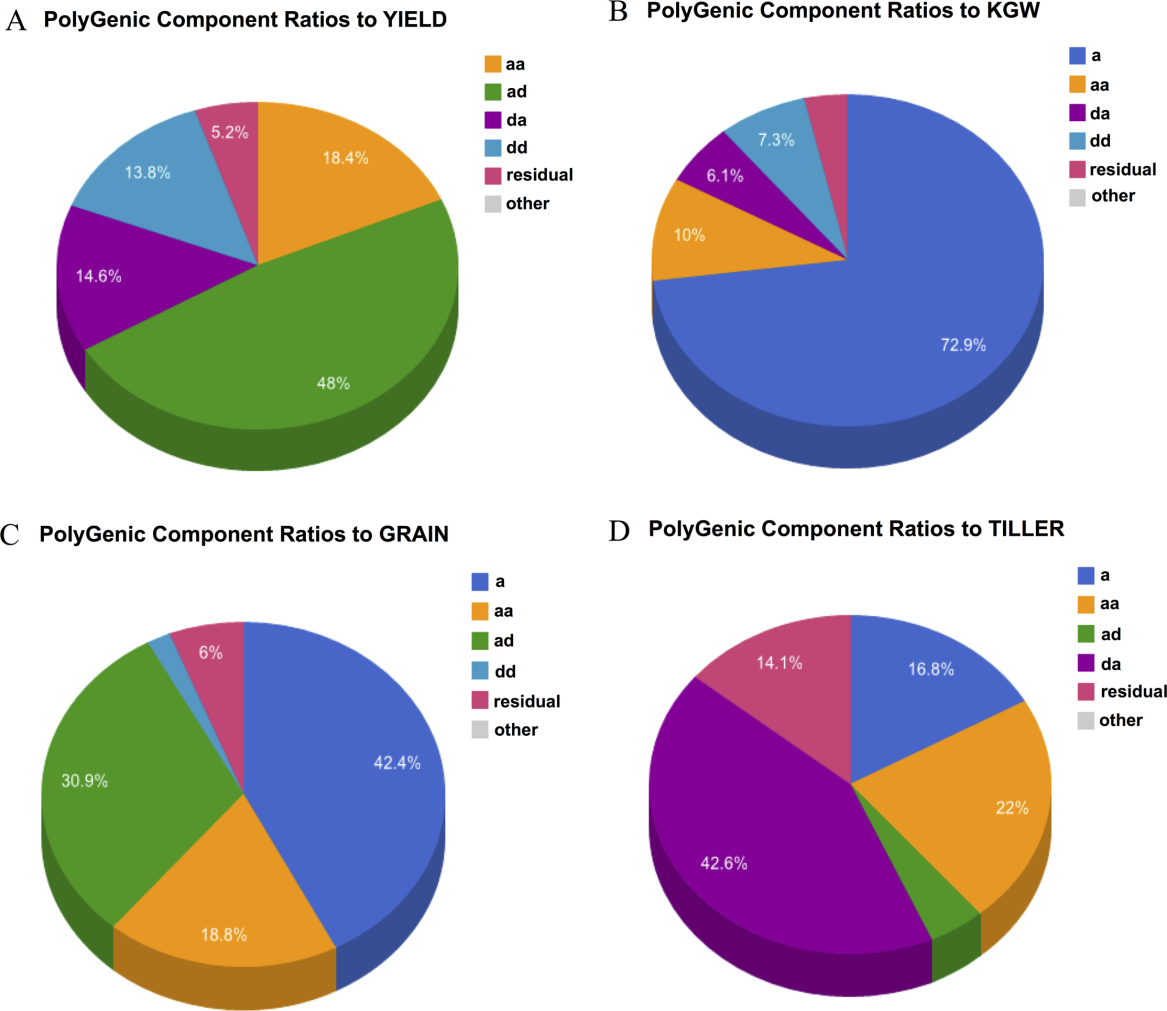
Pie charts illustrating the polygenic structure of the quantitative traits of rice. (A) YIELD, (B) KGW, (C) GRAIN, and (D) TILLER.

Based on the results of polygenic variance component analyses, the polygenic structure of a target quantitative trait can be dissected. Pie charts illustrating the polygenic variance component ratios for the traits YIELD, KGW, GRAIN, and TILLER are shown in **Fig 3**. Substantial differences between the polygenic structures were observed for the different traits. For example, additive genetic variance accounted for 73% of trait variance for KGW, whereas epistatic genetic variance was the major contributor to variance for YIELD.

**Fig 4:**
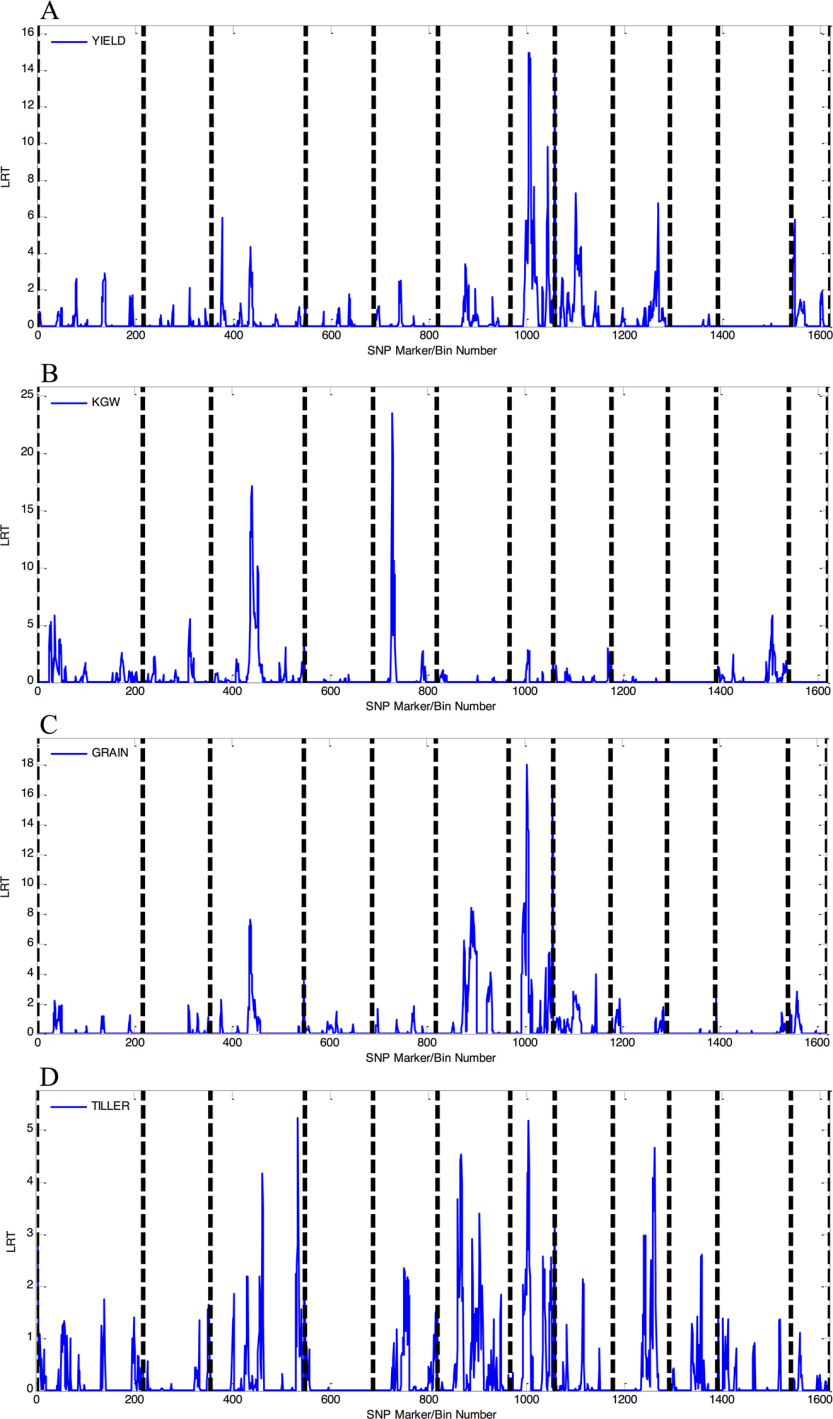
Plot of main-effect LRT results for quantitative traits with the markers/bins distributed across the complete rice genome. (A) YIELD, (B) KGW, (C) GRAIN, and (D) TILLER. Dashed lines distinguish the 12 chromosomes and corresponding marker/bin numbers for the complete rice genome.

The 1D LRT distributions across all markers/bins were plotted based on the results of genome scanning for main-effect QTL mapping. Plots of the main-effect LRTs for traits YIELD, KGW, GRAIN, and TILLER across the complete rice genome are shown in **Fig 4**. The LRT statistic can be used to declare the statistical significance for each marker/bin, herein, if we set a statistic threshold which also called p value, the marker/bin above the threshold suggest an association with the trait, further, the marker/bin under a narrower peak means a higher resolution and indicates a more specific association to the trait.

**Fig 5:**
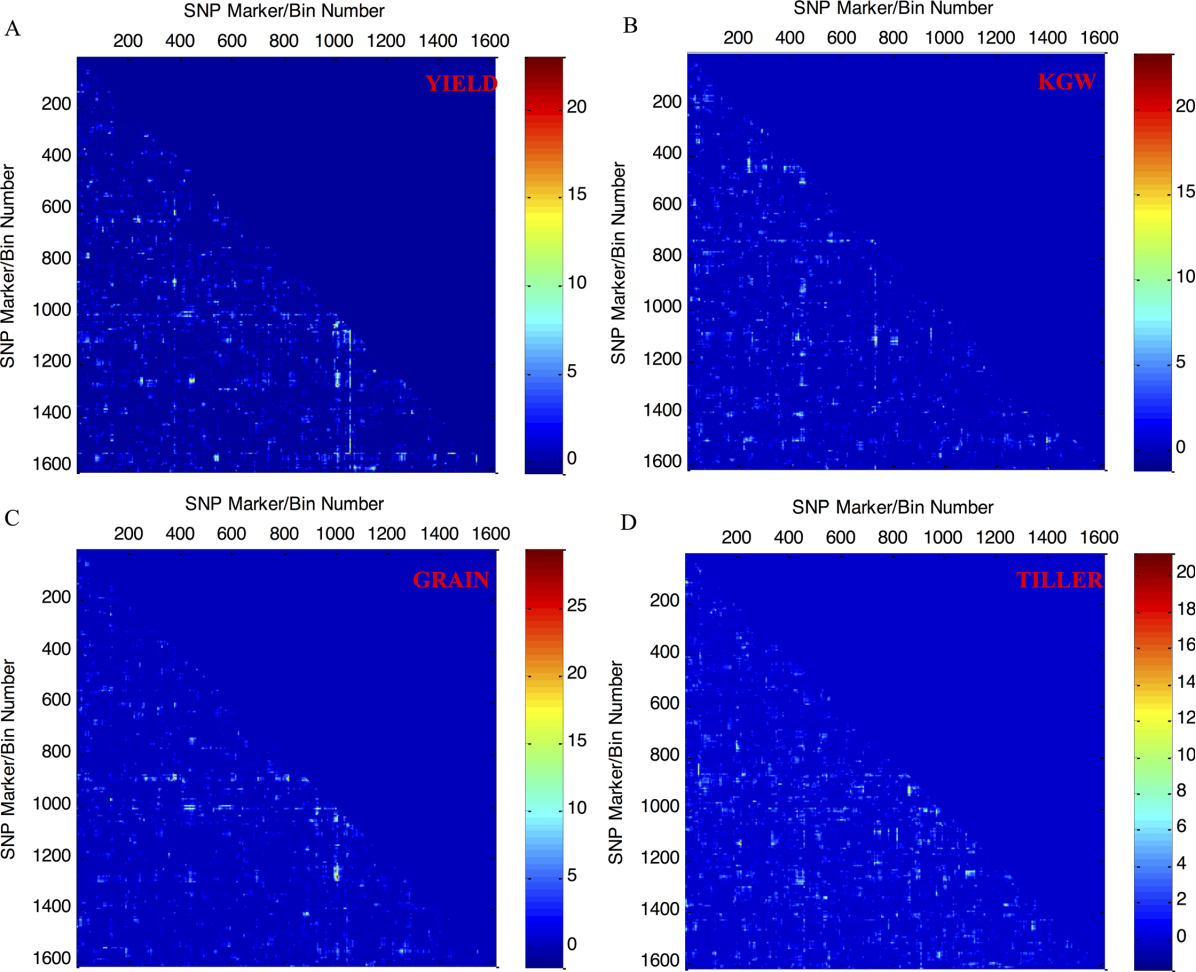
Two-dimensional illustration of epistatic-effect LRTs for quantitative traits with marker/bin pairs distributed across the complete rice genome. (A)YIELD, (B) KGW, (C) GRAIN, and (D) TILLER.

The 2D LRT distributions across all marker/bin pairs were also calculated based on the results of genome scanning for epistatic-effect QTL mapping. **Fig 5** shows the 2D epistatic-effect LRTs for the traits YIELD, KGW, GRAIN, and TILLER across the complete rice genome. Due to the symmetrical nature of the data, only a lower triangular matrix is shown for each trait instead of the entire square matrix for all of the $\frac{m(m-1)}{2}$ possible combinational marker/bin pairs. The 2D LRT statistic can be used to declare the statistical significance of each marker/bin pair, herein, a statistic threshold can be set, the marker/bin pair above the threshold suggests an association with the trait.

In summary, although the overall polygenic structure of several hybrid rice target traits can be obtained based on the data illustrated in **Fig 3**, greater detail regarding the genetic composition of these traits can be obtained from the 1D main-effect LRT data illustrated in **Fig 4** and the 2D epistatic-effect LRT data illustrated in **Fig 5**. The high-intensity profile peaks in **Fig 4** suggest that genetic loci are associated with the target traits examined in this study, for example, in **Fig 4(B)**, two high-intensity profile peaks located in chromosome 3 and chromosome 5 are two predominant genetic loci for trait KGW. Similarly, the high-intensity pixel points in **Fig 5** suggest that a marker/bin pair is associated with each of the target traits, for example, the vertical scale bars in in **Fig 5(A)** represented by a number of pixel points with comparatively high intensities and vertically scattered between marker/bin 1000-marker/bin 1600, suggest that there are numerous genetic marker interaction pairs. These pairs could be used to construct a more complex marker-marker interaction/regulation network for the trait of YIELD.

## Performance Analysis and Discussion

PEPIS was dedicatedly developed for epistatic genetic estimation. In generally, it has four independent modules including kinship matrix calculation, polygenic component analysis, genome scanning/mapping for main and epistatic effects. Currently, several software tools, such as TASSEL [26], GCTA [27,28], PLINK [28], etc. have been successfully developed for genome wide association mapping and complex trait analysis. These tools also need to calculate the kinship [26] / genetic relationship [27,28] matrix at first, and then estimate the genetic variances that can explain the phenotypic variances. In this regard, they have some similar functionalities as the PEPIS. However, all these tools are based on an additive linear model and ignore the epistatic effect. They calculated only one additive kinship matrix, and produced only a 1D likelihood-based association mapping. Comparatively, PEPIS is based on a full polygenic linear model. Therefore, PEPIS needs to calculate six polygenic kinship matrices, and produces both 1D and 2D likelihood based association mapping. The incorporation of epistatic effects results in a huge number of genetic epistatic effects to be estimated and presents a significant increase in computational burden.

Although PEPIS is equipped with a parallel strategy in a distributed Linux computing cluster, it is useful to perform a benchmark evaluation on the computation performance of PEPIS at different data scale. To accomplish this, we specifically generated a series of simulated data sets by varying bin/marker numbers and sample size. These simulated data sets are available in **S1 File**, which include 11 sub directories and each contains three '.txt' files, corresponding to the genotypic additive Z Matrix, dominance W Matrix, and vector of phenotypic values. The additive Z Matrix and dominance W Matrix are randomly generated but constrained by formula 1. We submitted each of the 11 data sets to PEPIS and recorded the running time for kinship matrix only and the whole running time for epistatic effect estimation and association mapping. Two scenarios of the simulation experiment were considered, and one was to fix the number of bins but vary the sample size (the number of individuals), the other was the opposite. The running times for kinship matrix calculations are shown in **S1 Table** and the overall running times for epistatic effect estimation and association mapping are shown in **S2 Table**.

From **S1 Table**, we can see that 1) the running time for calculating the main effect kinship matrix increases moderately with the increase of the number of bins; and 2) the running time for calculating the entire set of polygenic kinship matrices increases intensely with the increase of sample size and number of markers/bins. Such observations are consistent with our previous complexity analysis for kinship matrix calculation. The computational burden for main effect kinship matrix calculation is on the order of *O*(*mn*^2^), while the computational burden for epistatic effect kinship matrix calculation is on the order of *O*(*m*^2^*n*^2^).where *m* and *n* are the number of markers/bins and the sample size, respectively.

From **S2 Table**, we observe the followings: 1) the running time for epistatic effect estimation and association mapping increases intensely with the increase of the sample size and the number of markers/bins; and 2) the running time increases faster with the increase of sample size compared with the increase of bin number. Our in-depth investigation revealed that the module for polygenic variance component analysis takes significant amount of time when the sample size is very large, because the module is essentially an optimization procedure for a seven-parameter log-likelihood estimation, which takes the six polygenic kinship matrices as a whole input and cannot be parallelized.

It is well known that increasing the marker density and the sample size can further increase the resolution of QTL mapping and reduce the uncertainty of inferred genotypes. However, the high density markers can result in a huge number of marker pairs for epistasis detection. In PEPIS, the module for polygenic variant component analysis is still a bottleneck if the sample size is more than 5,000.

In summary, with the efficient algorithm implementation in C/C++ and deployment of parallel strategy, PEPIS has a powerful computational capability and is able to carry out epistatic effect analysis and association mapping when the sample size in the scale of several thousands and the number of markers/bins in the scale of twenty thousand. At these scales, it would require several years to be completed using the original prototype R programs [16].

## Availability and Future Directions

The PEPIS pipeline, the source code and the test data are freely available at http://bioinfo.noble.org/PolyGenic_QTL/. We are committed to maintaining and improving the specific function modules per user comments and suggestions.

The current version of the PEPIS can be configured to perform kinship matrix calculations, polygenic component analyses, the 1D LRT estimations for main-effect QTL mapping, and the 2D LRT estimations for epistatic-effect QTL mapping upon submission of the coded genotypic and phenotypic data. However, a more user-friendly and efficient visualization of the input genotypic data and the analysis results returned at each step would be very useful. We are therefore planning to develop a visualization engine that will allow for more efficient display of the input genotypic data and the polygenic QTL mapping results returned at each analysis step. Furthermore, the high intensities for a number of the pixel points shown in **Fig 5** are suggestive of the presence of marker interaction pairs that correspond to a biologically meaningful gene regulatory network. Therefore, we are also planning to develop an LRT (p value)-based genetic statistical network analysis module that will be incorporated into our publically available high-performance expression-based gene regulatory network analysis web-server (http://plantgrn.noble.org/GPLEXUS/) following validation [29].

Difficulties associated with handling high dimensional SNP data and the inability to estimate epistatic effects constitute a significant challenge in GWAS. Reducing the dimensions of SNP data based on biological information is critical and as such should be the first fundamental step in estimating epistatic effects. Xu pioneered a groundbreaking methodology for inferring breakpoints using high density SNP marker data from bi-parental populations and constructed a bin-based genetic marker data [30]. The segregation patterns are identical for all original SNP markers within a bin, and each bin in turn is considered to be a synthetic marker.

Binning markers in bi-parental populations is straightforward [30]. If the number of bins is still very large due to large sample sizes, one can combine several consecutive bins into a larger bin. As long as the number of these artificially created larger bins is small enough to be handed by the epistatic model, they can be used as “synthetic markers” for epistatic mapping. Wei and Xu[31] binned markers for multi-parent advanced generation inter-cross (MAGIC) populations for QTL mapping. They used the R software “Happy” to infer the parental origin of each marker for each individual and eventually binning consecutive markers with the same parental origins. For random populations, binning markers may be very difficult. However, we may use linkage disequilibrium (LD) value to define “bins”. Consecutive markers with LD larger than a threshold can be combined together and analyzed as a single bin. Such bins are better called LD blocks. Our PEPIS can take any numerical genotypic values as input files, regardless whether the genotypic values are defined as bins or original markers. One of our long term goals of the project is to incorporate a binning function into the pipeline so that the program can directly handle the original SNP data, leading to more practical applications of the PEPIS for epistatic analyses.

## Supporting Information

S1 File The simulated data at various dimensions with different sample sizes and different numbers of bins. Eleven sub directories are included, and each contains three '.txt' files corresponding to the additive genotypic Z Matrix, the dominance W matrix, and the phenotypic vector.
(ZIP)

S1 Table The PEPIS running time for kinship matrix calculation using the simulated data at various dimensions. Two scenarios are tested corresponding to A) Fixing sample size at 1000 while varying the number of bins from 1,000 to 40,000; and B) Fixing the number of bins at 1,000 while varying the sample size from 1,000 to 40,000.
(PDF)

S2 Table The PEPIS running time for estimating the epistatic effect in PEPIS using the simulated data at various dimensions. Two scenarios are tested corresponding to A) Fixing the sample size at 1,000 while varying the number of bins from 1000 to 20,000; and B) Fixing the number of bins at 1,000 while varying the sample size from 1,000 to 10,000.
(PDF)
