# Supplementary material for: PEPIS: A Pipeline for Estimating Epistatic Effects in Quantitative Trait Locus Mapping and Genome-Wide Association Studies
Source: PLoS Comput Biol. 2016 May 25;12(5):e1004925. doi: 10.1371/journal.pcbi.1004925 (PMC4880203; doi:10.1371/journal.pcbi.1004925)
Supplement: S1 Table — Two scenarios are tested corresponding to A) Fixing sample size at 1000 while varying the number of bins from 1,000 to 40,000; and B) Fixing the number of bins at 1,000 while varying the sample size from 1,000 to 40,000. (PDF) [file pcbi.1004925.s002.pdf]

S1 Table. The PEPIS running time for computing main effect kinship matrix (Ka, Kd) and full polygenic kinship matrix using the simulated data at various numbers of bins and individuals.

A. Fixing the number of Individuals at 1,000 while varying the numbers of bins from 1,000 to 40,000.

		Running time (seconds) for computing	
		Ka, Kd	Ka, Kd, Kaa, Kad, Kda, Kdd
Individual=1,000	Bin=1,000	87	66
	Bin=2,000	88	146
	Bin=4,000	95	345
	Bin=1,0000	322	2,056
	Bin=20,000	419	7,262
	Bin=40,000	545	29,322

B. Fixing the numbers of bins at 1,000 while varying the numbers of individuals from 1,000 to 40,000.

		Running time (seconds) for computing	
		Ka, Kd	Ka, Kd, Kaa, Kad, Kda, Kdd
Bin=1,000	Individual=1,000	57	63
	Individual=2,000	70	130
	Individual=4,000	158	427
	Individual=10,000	739	2,453
	Individual=20,000	2,763	9,710
	Individual=40,000	1,1131	39,024
