# Supplementary material for: PEPIS: A Pipeline for Estimating Epistatic Effects in Quantitative Trait Locus Mapping and Genome-Wide Association Studies
Source: PLoS Comput Biol. 2016 May 25;12(5):e1004925. doi: 10.1371/journal.pcbi.1004925 (PMC4880203; doi:10.1371/journal.pcbi.1004925)
Supplement: S2 Table — Two scenarios are tested corresponding to A) Fixing the sample size at 1,000 while varying the number of bins from 1000 to 20,000; and B) Fixing the number of bins at 1,000 while varying the sample size from 1,000 to 10,000. (PDF) [file pcbi.1004925.s003.pdf]

S2 Table. The total running time of PEPIS for the epistatic effect analysis using the simulated data at various numbers of bins and individuals.

A: Fixing the numbers of individuals at 1,000 while varying the numbers of bins from 1,000 to 20,000.

		Epistatic Effect Analysis Running Time (seconds)
Individual=1,000	Bin=1,000	701
	Bin=2,000	1,140
	Bin=4,000	1,949
	Bin=10,000	7,901
	Bin=20,000	28,356

B: Fixing the numbers of bins at 1,000 while varying the numbers of individuals from 1,000 to 10,000.

		Epistatic Effect Analysis Running Time (seconds)
Bin=1000	Individual=1,000	701
	Individual=2,000	4,399
	Individual=4,000	35,885
	Individual=10,000	506,552
